# Enhanced Hydrogen Detection Based on Mg-Doped InN Epilayer

**DOI:** 10.3390/s18072065

**Published:** 2018-06-28

**Authors:** Shibo Wang, Xinqiang Wang, Zhaoying Chen, Ping Wang, Qi Qi, Xiantong Zheng, Bowen Sheng, Huapeng Liu, Tao Wang, Xin Rong, Mo Li, Jian Zhang, Xuelin Yang, Fujun Xu, Bo Shen

**Affiliations:** 1 State Key Laboratory of Artificial Microstructure and Mesoscopic Physics, School of Physics, Peking University, Beijing 100871, China; 1501210114@pku.edu.cn (S.W.); chen_zhaoying@pku.edu.cn (Z.C.); wangping210049@163.com (P.W.); zxt1001@live.cn (X.Z.); subway715@pku.edu.cn (B.S.); liuhp999@163.com (H.L.); cwwangtao@163.com (T.W.); rongxin@pku.edu.cn (X.R.); xlyang@pku.edu.cn (X.Y.); fjxu@pku.edu.cn (F.X.); bshen@pku.edu.cn (B.S.); 2 Collaborative Innovation Center of Quantum Matter, Beijing 100871, China; 3 Dongguan Institute of Opto-Electronics, Peking University, Dongguan 523808, China; qiqi@sinoagg.com; 4 Microsystem & Terahertz Research Center, No 596, Yinhe Road, Shuangliu, Chengdu 610200, China; limo@mtrc.ac.cn (M.L.); zhangjian@mtrc.ac.cn (J.Z.)

**Keywords:** InN, Mg-doping, hydrogen sensor

## Abstract

It is a fact that surface electron accumulation layer with sheet electron density in the magnitude of ~10^13^ cm^−2^ on InN, either as-grown or Mg-doped, makes InN an excellent candidate for sensing application. In this paper, the response of hydrogen sensors based on Mg-doped InN films (InN:Mg) grown by molecular beam epitaxy has been investigated. The sensor exhibits a resistance variation ratio of 16.8% with response/recovery times of less than 2 min under exposure to 2000 ppm H_2_/air at 125 °C, which is 60% higher in the magnitude of response than the one based on the as-grown InN film. Hall-effect measurement shows that the InN:Mg with suitable Mg doping level exhibits larger sheet resistance, which accords with buried p-type conduction in the InN bulk. This work shows the advantage of InN:Mg and signifies its potential for sensing application.

## 1. Introduction

As the demand for new energy market continues to rise, hydrogen is increasingly favored by many parties as an efficient and environmentally friendly fuel. However, hydrogen suffers from storage and transportation danger because it is prone to leak and easy to be ignited and explode even at low concentration (>4%) in the air. Unfortunately, it is also difficult to detect the leakage because of the colorless and odorless feature of hydrogen. Thus, the development of hydrogen sensors is increasingly important [1]. In fact, the study on hydrogen sensors has been continued for several decades [2,3,4,5,6,7]. Recently, hydrogen sensors based on III-nitrides have attracted more and more attention due to their good thermal stability and durability in a harsh environment [8,9,10,11]. Those hydrogen sensors are mostly based on an AlGaN/GaN heterostructure since two-dimensional electron gas (2DEG) is formed at the interface between AlGaN and GaN due to the large band offset between AlGaN and GaN and the internal electric field is driven by spontaneous and piezoelectric polarization, which are sensitive enough because the AlGaN barrier is thin and the drain current response can be amplified through a functionalized gate electrode.

**Figure 1:**
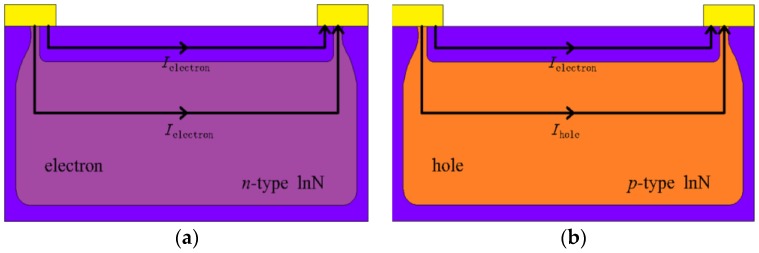
Schematic diagrams of conduction mechanism for n-type (**a**) and p-type (**b**) InN sensors.

In the III-nitrides material family, indium nitride (InN) exhibits a novel phenomenon of surface electron accumulation layer, manifested by a high sheet electron density in the order of 10^13^ cm^−2^ occurring within 5 nm from the surface [12,13,14]. Such electronic properties have been theoretically proven to be beneficial for sensor application [15,16], leading to the realization of InN-based field-effect transistors for sensing application with high response [17,18,19,20,21]. However, as-grown InN is usually strong n-type, in which the contribution of bulk conductivity cannot be neglected, as is shown in Figure 1a, resulting in a limitation to sensor sensitivity. On the other hand, since the surface electron layer also exists in the Mg-doped buried p-type InN film, the p-type InN should be a better candidate than the as-grown n-type InN. In the usual case, there should be a depletion layer between the surface electron accumulation and the buried p-type layer, making the surface layer isolated from the bulk, and this provides us with a unique idea for sensing application [22,23]. As is shown in Figure 1b, given that the electron density in the accumulation layer is high and its mobility is much larger than the hole mobility, the contribution of surface electrons to the whole conductivity dominates that from the holes [22,24,25,26]. Unfortunately, to the best of our knowledge, there is the scarce report on gas sensing using InN film with buried p-type conduction until now.

In this article, we have fabricated hydrogen sensors by using Mg-doped InN films (InN:Mg), which are grown by molecular beam epitaxy. The hydrogen sensor exhibits a resistance variation ratio of 16.8% with response/recovery times of less than 2 min under exposure to 2000 ppm H_2_/air at 125 °C, which is 60% higher in the magnitude of response than that based on as-grown InN film. We believe that the use of InN with a buried p-type layer can effectively dwarf the contribution of bulk conductivity and optimize the surface-to-volume ratio, resulting in a large enhancement in sensor response.

## 2. Materials and Methods

**Figure 2:**
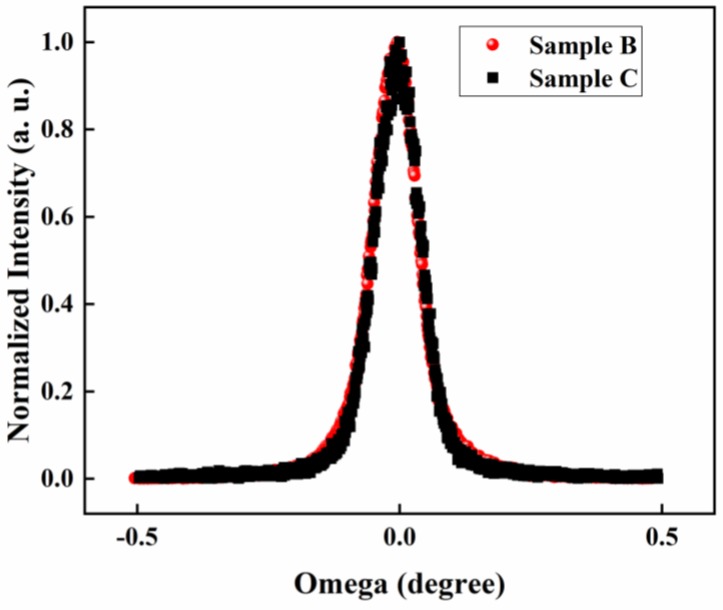
The XRD rocking curves of the two samples measured across (002) plane.

InN films were grown on sapphire substrates by plasma-assisted molecular beam epitaxy (PA-MBE). GaN layer with a thickness of 4 μm was grown first by metal-organic chemical vapor deposition (MOCVD) and was used as a template for InN growth. After regrowth of a 100-nm-thick GaN buffer layer on the GaN template, undoped and Mg-doped InN were directly grown on the GaN layer under slightly In-rich condition [27]. The typical thickness of InN is about 15 nm and the Mg cell temperature is varied from 220–280 °C during InN:Mg growth. Detailed sample parameters are listed in Table 1. The sheet resistance of as-grown InN and InN:Mg samples are extracted from Hall-effect measurement, which is also listed in Table 1. To confirm the quality of Mg-doped InN samples, X-ray diffraction (XRD) measurement on as-grown InN (sample C) and Mg-doped InN (sample B) was also performed. The XRD rocking curves of the two samples measured across (002) plane are shown in Figure 2 in which the FWHM of as-grown InN is 375 arcsec and the FWHM of Mg-doped InN is 363 arcsec. It is observed that the XRD rocking curves of the two samples measured across (002) plane are very close, indicating that the Mg doping does not significantly affect the crystal quality.

**Figure 3:**
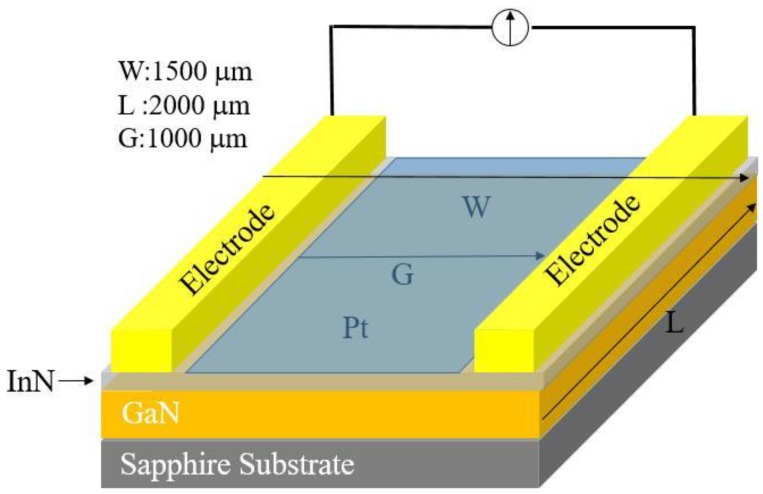
Schematic structure of the hydrogen sensor based on Mg-doped InN.

These samples were treated with HCl solution to remove indium droplets on the surface before device fabrication. The schematic structure of the hydrogen sensor is shown in Figure 3, where the device is comprised of a pair of Ohmic contact electrodes of Au/Al/Ti (50 nm/200 nm/50 nm) composite and a sensing window (1.0 mm in width × 2.0 mm in length) defined by a 10-nm-thick Pt film between the electrodes on the InN surface. All metal films were deposited in an electron-beam evaporation system and patterned by a photolithographic liftoff process.

The resistance between the two electrodes was measured in response to hydrogen exposure at different temperatures (25 °C, 80 °C, and 150 °C) and at various H_2_ concentrations (100–2000 ppm H_2_/air). The hydrogen concentrations are modified by adjusting the ratio of the flow rates of two gas channels, i.e., hydrogen and synthetic air. Since the background resistance of each sensor is different from others, we define our response as resistance variation ratio:

(1) $$\mathrm{Response}=\left|R_{H_{2}}-R_{\mathrm{bg}}\right|/R_{\mathrm{bg}}\times 100\%$$

In which $R_{H_{2}}$ means the resistance exposed under hydrogen and $R_{\mathrm{bg}}$ is the background resistance of each sensor. 

## 3. Results and Discussion

**Figure 4:**
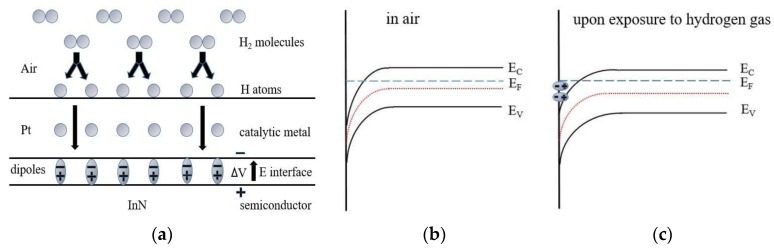
A schematic diagram of hydrogen adsorption process. (**a**) Formation of a dipole layer, formed by hydrogen atoms trapped at the interface of catalytic metal and semiconductor, causing a voltage shift. (**b**) The corresponding schematic energy band diagram of the studied device (**b**) at air and (**c**) under the introduction of hydrogen gas.

The model to explain the response to hydrogen exposure for Pt-semiconductor contact is based on the interaction between hydrogen-induced interface dipoles and two-dimensional electron gas (2DEG) in the semiconductor [28], which is already shown in Figure 4a. The atomic hydrogen dissociated through the catalytic action of Pt diffuses and then becomes being adsorbed at the Pt/semiconductor interfaces, inducing an interface dipole layer and thus leading to a modulation of the near-surface carrier concentration [28,29]. Figure 4b,c show the corresponding energy-band diagram of our sensors at equilibrium and under the introduction of hydrogen gas. Apparently, a barrier height modulation (decrease) at InN surface is observed once hydrogen gas is introduced. This change will cause the related modulation of two-dimensional electron gas (2DEG). Therefore, under the introduction of hydrogen gas, the change of electrical properties could be used to calibrate hydrogen concentration.

**Figure 5:**
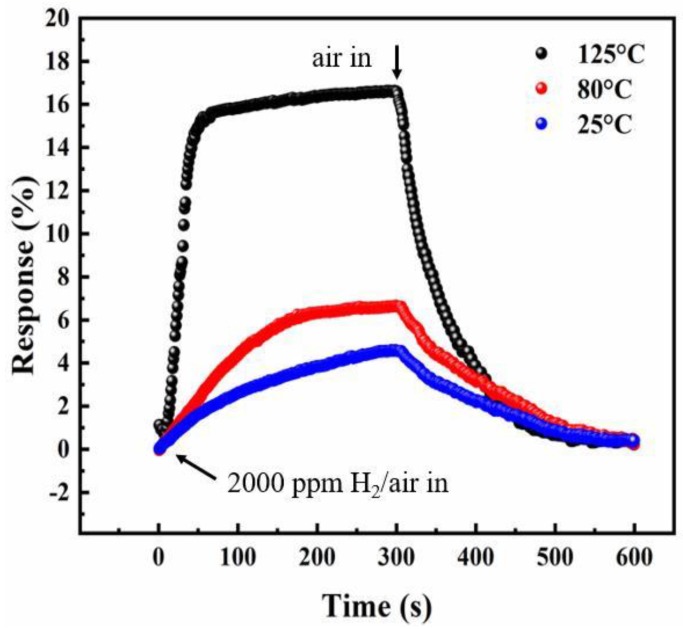
Response as a function of exposure time for sample A under the exposure to 2000 ppm H_2_/air and then recovery in the air at different temperatures from 25 °C to 125 °C.

Figure 5 shows the response of the InN sensor based on InN:Mg (sample A) upon exposure to 2000 ppm H_2_/air at various temperatures from 25 °C to 125 °C. The response and recovery times of the sensor can be defined as the elapsed times of the relative resistance change from 10% to 90% and from 90% to 10%, respectively [18]. The sensor exhibits a response of 4.5% under 2000 ppm H_2_/air at 25 °C, while the response and recovery times are both about 5 min. As the operating temperature is up to 125 °C, the response is enhanced to about 15% with a much shorter response and recovery time, less than 2 min. This is because the high temperature can increase the catalytic dissociation rate of molecular hydrogen (H_2_) or diffusion rate of atomic hydrogen into the Pt/InN interface [30]. It can be seen that our sensor did not have any response in the case of synthetic air only, which further confirmed that the sensing signals completely came from hydrogen gas. Therefore, the cross sensitivity is unnecessary to be check for the applications in the air atmosphere, such as leakage monitoring of H_2_ during transportation.

**Figure 6:**
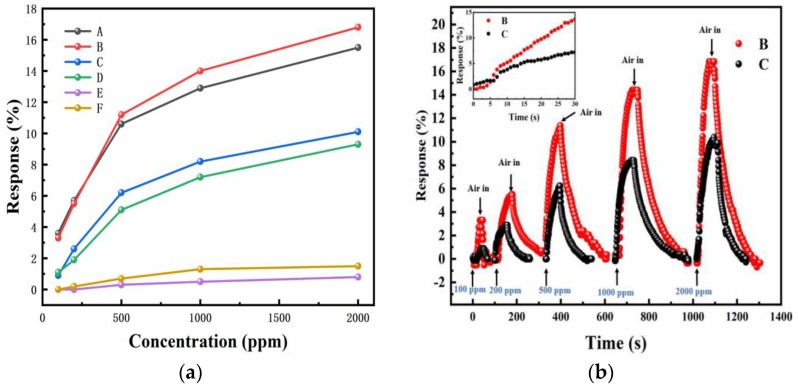
Resistance variation as a function of hydrogen concentrations for Mg-doped InN sensors at 125 °C at different Mg cell temperatures. (**a**) The response of all samples as a function of gas atmosphere. (**b**) The response curve of sample B and C. The inset shows their transient response in first 30 s upon exposure to 2000 ppm H_2_/air, almost linear with respect to time.

Then, the effect of Mg-doping on hydrogen sensing was characterized by measuring the response of the Mg-doped InN and as-grown InN sensors at 125 °C and at hydrogen concentration from 100 to 2000 ppm. The response of samples with different Mg cell temperatures and different gas atmospheres were shown in Figure 6a. According to previous reports and the sample growth conditions [31,32,33,34,35], there are three regions with increasing Mg cell temperature (T_Mg_): Region I (T_Mg_ < 220 °C) and III (T_Mg_ > 260 °C) refers to the slightly-doped and overdoped regime, where the samples are n-type. Region II (220 °C < T_Mg_ < 260 °C) usually shows the evidence of p-type conduction in the bulk. It is observed that for samples A and B in Region II, their response is much higher than that of the sensor based on unintentionally doped InN, i.e., sample C. Especially under 2000 ppm H_2_/air at 125 °C, the response of sample B is about 16.8%, even 60% higher than that of sample C (10.1%). For sample D in the Region I, in which InN is lightly doped, its response is close to sample C, and when T_Mg_ is 280 °C, in Region III, the response of sample E only shows little response, even much lower than sample C. For sample F in Region II with a much larger thickness, it also has a weak response. Figure 6b shows the response curve of sample B and C, and the inset shows their transient response in first 30 s upon exposure to 2000 ppm H_2_/air, which is similar to the unintentionally doped InN sensor previously reported [18], almost linear with respect to time.

It has been reported that the InN with suitable Mg-doping shows a buried p-type behavior, where the directly probed result by Hall effect measurement shows n-type conduction due to the electron domination in mobility-weighted contributions of both types of carriers [32]. Thus, a standard multilayer model can be used to explain the advantage of InN:Mg, in which the sensor is divided into two regions (surface and bulk), as is shown in Figure 1a,b. We assume that the influence of proximity between these two layers could be negligible and they are shorted at the contacts [32,36]. The relationship for the multilayer model is [27,31]:

(2) $$\sigma_{\mathrm{total}}={\mathrm{en}}_{H}\mu_{H}={\mathrm{en}}_{s}\mu_{s}+{\mathrm{en}}_{b}\mu_{b}d,$$

(3) $$n_{H}\mu_{H}{}^{2}=n_{s}\mu_{s}{}^{2}+n_{b}\mu_{b}{}^{2}d,$$

where σ_total_ is the total sheet conductivity; n and μ are carrier density and carrier mobility, respectively; the subscript s and b represent the surface electron accumulation layer and the bulk, respectively; d represents the thickness of bulk; and H represents the direct measurement value.

In region II, the concentration of the ionized Mg is higher than that of the ionized donors, leading to the buried p-type doping [31]. Since the hole mobility in the buried p-type InN is much smaller than that of the electron [22,24,25,26], the contribution of bulk conductivity (${\mathrm{en}}_{b}\mu_{b}d$) is very low, which means the surface contribution (${\mathrm{en}}_{s}\mu_{s}$) is dominant. Thus, the total conductivity (σ_total_) for samples with p-type carriers in the bulk is much lower than others, as is shown in Table 1, in which samples A and B have the largest sheet resistance. Due to the low surface electron mobility [37] and its dominant role, the mobility of sample A and B probed directly by Hall Effect measurement are also much lower than that of sample C while at similar sheet concentration, which is consistent with previous report [23]. For the rest conditions, sample D is n-type because the doped acceptors cannot completely compensate the residual donors. Sample E is in region III, the over-doped case in which the excessive Mg reduces the formation energy of donor-like defects and complexes but increases the formation energy of acceptor-like ones, leading to a strong n-type conduction again [31,32]. For the n-type InN, the contribution of bulk conductivity becomes much larger since the majority carrier in bulk changes from hole to electron, making the total conductivity jump up, which means that surface contribution accounts for a lower proportion of the total conductivity. Given the dark current in buried p-type, InN should be much smaller and Mg doping does not have a much influence on the surface sensitivity [38,39]; InN sensors with a buried p-type layer are more sensitive than that based on strong n-type InN. However, it should also be noted that the bulk contribution also increases with the thickness. As is shown in Figure 6a, although the Mg cell temperature of sample F was 250 °C, it still has a low response because of its large thickness, resulting in a low surface-to-volume ratio.

Since the electron accumulation layer of InN is located underneath the surface, i.e., the gas-sensing window, the interaction between the dipoles and the near-surface electrons should be stronger than that in HEMT-based hydrogen sensors which have an intermediate dielectric layer between the interface and the 2DEG. However, compared to the sensors based on HEMT structure previously reported, our p-type InN sensors do not show obviously improved performance, which is still in the same order of magnitude [8,40,41]. This is because there is a large amount of threading dislocation running through the film, connecting the surface layer with the bulk and even the electron accumulated between the interface of InN and GaN. Thus, the surface electron accumulation is still affected by other paralleled conducting channels. The performance degradation after almost one month is also observed. With time, not only the response decreases but also the maximum hydrogen concentration that the sensor can detect becomes much lower. A possible reason is that InN surface becomes contaminated by oxygen. When InN sensing layer comes into air ambient, a thin layer of indium oxide will form on the surface of the InN sensing layer due to adsorption of oxygen, which can ionize in many forms like $O_{2}^{-}$, $O^{-}$ and $O^{2-}$ [42]. The adsorbed oxygen ions can block available surface adsorption sites of platinum for hydrogen absorption or even react with hydrogen on the surface, lowering the concentration of atomic hydrogen at the metal/semiconductor interface [43]. As was mentioned previously, our sensors perform better when working at high temperature, which also enhances the oxygen ionization [42]. So, there must be a balance between good performance and performance degradation. On the other hand, the electrons transferred from bulk InN to the chemisorbed oxygen will lead to an accumulation of negative charges on the surface, neutralizing the positively charged donor-type surface states, causing a reduction of the electron accumulation [38,44] and dwarfing the surface sensitivity. In addition, the influence of humidity cannot be ignored. It has been reported that Pt adsorbs H_2_O and catalytically dissociates H_2_O to form OH molecules with the assistance of surface chemisorbed atomic oxygen [43]. Those OH molecules can have a reaction between H and OH into the formation of H_2_O, whose consumption of Hydrogen cannot be neglected.

These factors discussed above can have the serious impact on sensor performance. An alternative solution is to use high-resistance substrate and to introduce professional package process to enhance its stability in the atmosphere, which will be investigated later.

## 4. Conclusions

The hydrogen sensors based on Mg-doped InN films grown by molecular beam epitaxy have been demonstrated. The sensors based on Mg-doped InN perform higher response with larger sheet resistance, where the resistance variation is 16.8%, almost 60% higher than that of the sensor on as-grown InN under exposure to 2000 ppm H_2_/air at 125 °C. Degradation of the sensor performance was observed, which is probably related to the chemisorbed oxygen on the surface. Despite this degradation, the Mg-doped InN with a buried p-type conduction is still an excellent candidate for sensing applications.

## Figures and Tables

**Table 1:** The parameters and Hall measurement results of all samples.

Sample	Mobility (cm^2^/(V × s))	Ns (10^13^/cm^2)^	Rs (Ω/Square)	Mg Cell Temperature (°C)	Thickness (nm)
A	91	−7.9	863.7	250	15
B	107	−6.5	880.2	250	15
C	193	−5.6	578.6	——	15
D	227	−6.4	430.3	220	15
E	138	−31.0	147.6	280	15
F	127	−19.5	251.1	250	500
